# Beyond Standard Radiotherapy: an AI-Driven Framework for Personalized Radiotherapy with a Four-Step Classification in Head and Neck Squamous Cell Carcinoma (HNSCC) Patients

**DOI:** 10.1007/s11912-025-01730-x

**Published:** 2025-11-01

**Authors:** Yongxin Guo, Eric O. Aboagye, Dorothy M. Gujral

**Affiliations:** 1 Department of Surgery and Cancer, Faculty of Medicine, Imperial College London, London, W12 0NN United Kingdom; 2 Department of Radiotherapy, Imperial College Healthcare NHS Trust, London, W6 8RF United Kingdom

**Keywords:** Personalized radiotherapy, Head and neck squamous cell carcinoma, AI-driven radiotherapy, Spatiotemporal imaging, Radiomics

## Abstract

**Purpose of Review:**

Radiotherapy (RT) is a mainstay treatment strategy for patients with radically treatable head and neck cancer (HNC). Efforts to personalize RT have spanned decades, yielding variable results across different treatment stages. The purpose of this review was to assess the potential of AI-driven models to bridge personalized radiotherapy strategies.

**Recent Findings:**

Radiomics, an emerging imaging analytics approach, provides significant quantitative features that can predict survival outcomes, treatment responses, and radiation-induced toxicity. Radiomics-based models in the studies demonstrate promising predictive efficacy with a high C-index or area under the curve (AUC) exceeding 0.70.

**Summary:**

AI-driven multimodal and multitemporal imaging models can stratify patients and guide risk-adapted radiotherapy. A four-step AI-driven RT framework may provide a template for future randomized controlled trials, supporting more trustworthy evidence.

**Supplementary Information:**

The online version contains supplementary material available at 10.1007/s11912-025-01730-x.

## Introduction

Head and neck cancer (HNC) is becoming a more pressing health issue. According to the Global Cancer Statistics 2022 (GLOBOCAN), HNC accounts for 4.79% of cancer incidence and 4.94% of cancer mortality worldwide, ranking sixth amongst all cancers [1]. The distribution of HNC subsites exhibits a distinct geographic pattern. Beyond the well-established independent risk factor of tobacco use, infectious etiologies have emerged as increasingly important vulnerabilities in recent years—particularly human papillomavirus (HPV16) and Epstein–Barr virus (EBV). Head and neck squamous cell carcinoma (HNSCC) is the predominant subtype, consist of over 90% of HNC cases. Unlike the increased incidence of HNC, clinical outcomes have remained relatively stable, with five-years overall survival (OS) at approximately 50% [2]. Therefore, personalized precision therapy warrants further scrutiny.

Over the past decades, robust biomarkers have been sought to improve patient stratification, particularly in locally advanced HNSCC, where substantial heterogeneity often leads to suboptimal tumour control. Traditional biomarkers derived from pathology, serum, or liquid biopsy can capture certain risk-associated signatures, nevertheless, their reliance on a single spatial subregion or single temporal snapshot limits their ability to represent the full biological complexity of tumours. Radiomics offers a transformative alternative by enabling voxel-wise quantitative characterization of the tumour phenotype and the peritumoral microenvironment, thereby providing a multidimensional view of spatiotemporal heterogeneity. Radiomics-based AI models have demonstrated strong potential to refine predictive prognosis, guide risk-adapted treatment, and serve as a high-throughput pathway toward individualized radiotherapy strategies.

In this review, we do not reiterate the radiomics workflow or its application in diagnosis and auto-segmentation, as these topics have been extensively discussed previously. Instead, we summarize the standard radiotherapy strategy and current individualized treatment attempts, with particular emphasis on the potential role of radiomics in personalized radiotherapy for HNSCC patients. This promising approach may offer more advantages beyond standard treatments. Ultimately, we propose a “4-step classification” to stratify HNSCC patients throughout their comprehensive cancer management, which may provide a template for designing future randomized controlled trials based on AI models, with head-to-head comparison with standard treatment.

## Current Radiotherapy Strategy

Traditional Tumour, Nodal, Metastasis (TNM) staging is still the basis for treatment decision-making, emphasizing primary tumour extension, loco-regional lymph nodes invasion, and distance metastasis. For early-stage HNSCC patients (T1-2N0), radical radiotherapy (RT) and surgery yield similar long-term survival rates, with around 80% of patients remaining free of local recurrence and distant metastasis for 5 years [3, 4]. Certain subsites like nasopharyngeal cancer (NPC) [5], p16-positive oropharyngeal cancer (OPC) [6], and glottic cancer [4] may achieve better local control (LC) of over 90%. Nevertheless, due to the tumour heterogeneity, locally advanced cases show significant variability in clinical outcomes with standard care. Considering tumour infiltration and complicated adjacent anatomy, the choice of systemic therapy must be balanced amongst tumour control, treatment-related side effects, function preservation and aesthetic appearance. Synchronous chemoradiotherapy (CRT) is the cornerstone strategy for locally advanced patients, with dose escalation from low- to high- risk regions, with platinum-based monochemotherapy.

For patients unable to tolerate curative-intent treatment, particularly older adults with poor performance, palliative radiotherapy can yield a favorable objective response rate (ORR) and provide symptom relief [7]. In metastatic and recurrent HNSCC, salvage radiotherapy and reirradiation also play a significant role in improving local control at both primary and metastatic sites [8]. Moreover, advances in RT technology have introduced novel treatment techniques that combine dosimetric and radiobiological advantages, including proton radiotherapy (PRT), heavy ion therapy (HIT), spatially fractionated radiotherapy (SFRT), and ultra-high dose rate radiotherapy (FLASH-RT).

## Attempts Toward Personalized Radiotherapy

**Fig. 1:**
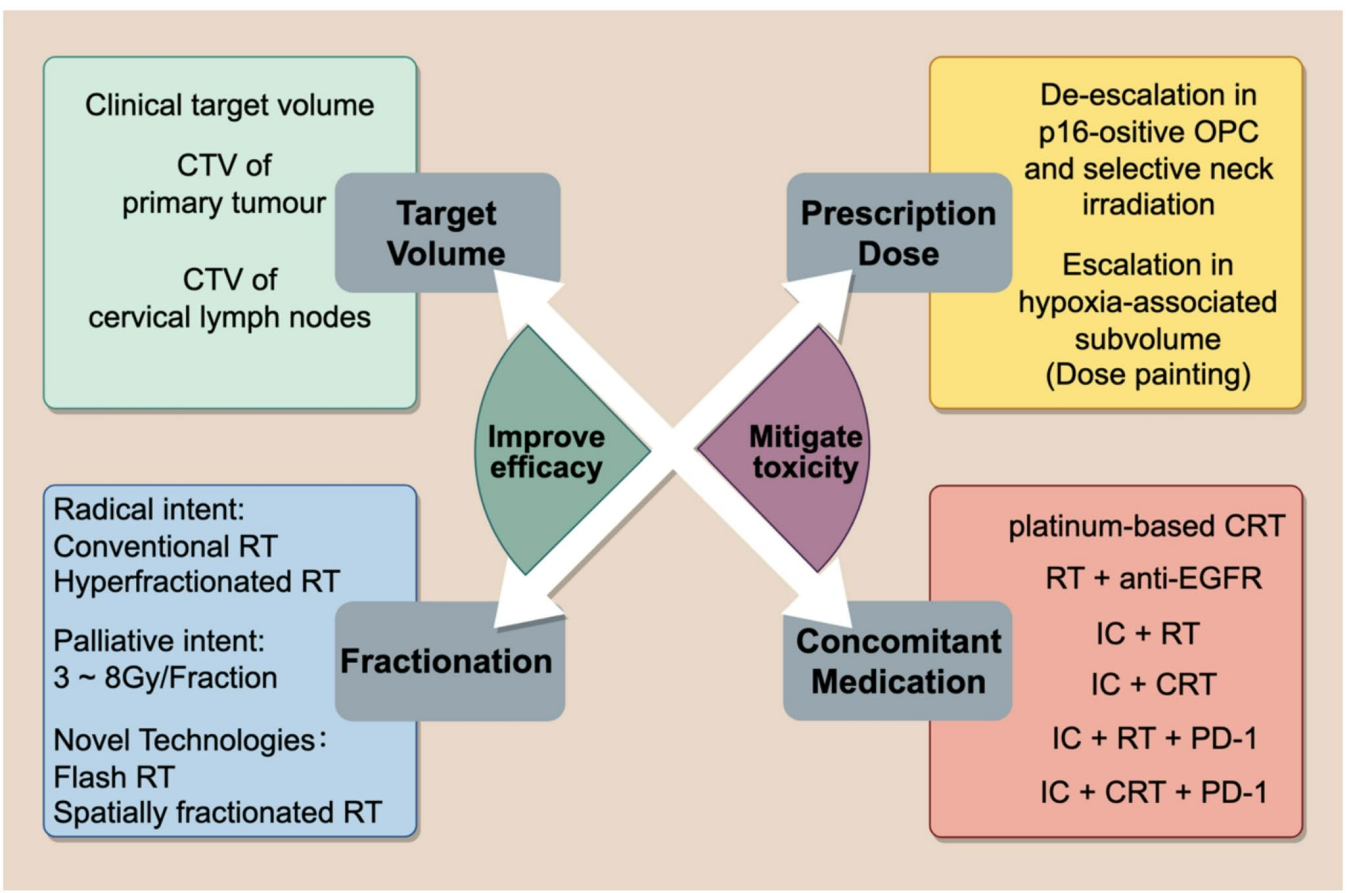
Attempts toward personalized radiotherapy. OPC, oropharyngeal cancer. Gy, Gray. RT, radiotherapy. CRT, concurrent chemoradiotherapy. anti-EGFR, epidermal growth factor receptor inhibitors. IC, induction chemotherapy. PD-1, programmed cell death protein 1 inhibitors

The primary objective of radiation oncologists is to improve the therapeutic ratio in an optimal manner, which improves radiotherapy efficacy and prevents or mitigates radiation-induced adverse events (rAEs). The target volume, dose fractionation, and prescription dose are crucial components of personalized radiotherapy, as shown in Fig. 1. However, comparisons between hyperfractionated RT and concomitant CRT [9], and controversial results of dose de-escalation strategies in HPV-associated OPC (e.g., the NRG-HN005 trial [10] and Quarterback trial [11]), indicate that firm conclusions cannot yet be drawn. Therefore, we place greater emphasis on personalized target volume.

Delineation of the clinical target volume (CTV) is significantly influenced by the definition of the high-risk regions. Typically, the primary tumour CTV should encompass the gross tumour volume (GTV) along with the surrounding tissue at risk for microscopic tumour infiltration. The maximal extension of microscopic disease from the edge of the primary tumour varies in surgical specimens, ranging from 3 to 12 mm, and is affected by the anatomical tumour subsite [12, 13]. The CTV margin from the GTV can vary across cancer institutions [14]. In 2018, an international consensus [15] that synthesized global guidelines proposed the concept of a “5 plus 5 mm expansion” CTV margin from the GTV, advocating a standard approach to reduce treatment variability. However, this approach does not guarantee that a “5 plus 5 mm margin” will encompass 100% of microscopic tumour extension or avoid unnecessarily covering normal tissues [16, 17]. Consequently, this results in some geometrical uncertainty for individual cases.

HNSCC patients who experienced tumour reduction after induction chemotherapy (IC) were recently shown to have the possibility of receiving to the reduced-volume RT, in EBV-related nasopharyngeal cancer patients from an endemic area [18]. Reduced-volume radiotherapy based on post-IC tumour volume showed noninferior 3-year locoregional relapse-free survival than pre-IC tumour volume irradiation, 91.5% vs. 91.2% respectively, with lower incidence of grade ≥ 3 radiation-related toxicities. Despite consensus on delineation of the neck node levels for HNC [19], elective nodal irradiation (ENI) is an alternative option for a tailored strategy. Involved-field irradiation (IFI) has been demonstrated to be effective non-small cell lung cancer (NSCLC) [20] and esophageal cancer [21], offering similar outcomes to ENI but with significant advantages on toxicity control. However, in HNSCC, there is a lack of clinical trials supporting reduced irradiation of the draining lymph node, except under highly selective conditions such as clinical N0-N1 disease or ipsilateral neck involvement [22]. Results-guided ENI strategies are still under investigation, including the PRIMO trial (NCT 05333523) [23] and SELET trial (NCT 05451004) [24], which are guided by sentinel lymph node biopsy pathology and lymph node mapping using single photon emission computed tomography, respectively. Indeed, HNSCC present a formidable challenge due to the complicated lymphatic network in cervical lymph nodes and the high likelihood of occult metastasis even at the pN0 stage [25]. HNSCC commonly spreads along the mucosa, interstitial space, lymphatic drainage and cranial nerve channel [26], resulting in a substantial risk of relapse if utilizing simple narrow irradiation fields.

## Radiomics

Radiomics, initially proposed by Lambin et al. in 2012, extracts large amounts of image features to indicate the spatial and temporal heterogeneity in a non-invasive way [27]. Conventional imaging relies on subjective visual interpretation by radiologists, providing anatomical information and to stage the tumour. Comparatively, radiomics provides a rather objective and quantitative approach to detect and distinguish abnormal features. It has seen a surge in applications across various medical fields, and maps digital medical images into quantitative data, offering clinical decision-support by generating imaging biomarkers. Radiomics integrates multimodal imaging, such as CT, MRI, PET, and ultrasound, with multitemporal imaging (pre-, during, and post- treatment) to select specific characteristic features, which could potentially improve the accuracy of diagnosis and outcome prediction. These radiomics features also reflect intratumoral and peritumoral heterogeneity, which is strongly linked to gene-expression patterns and tumour microenvironment [28, 29]. Several common genes implicated in the tumour occurrence, development and metastasis in HNSCC, such as p16, p53, EGFR and VEGF, have been demonstrated to show that the expression of these genes have strong correlation with functional imaging features [30].

## Potential Application of Radiomics in Personalized Radiotherapy

Modern radiotherapy practices rely on imaging data at each phase of comprehensive cancer management, including tumour diagnosis and staging, radiotherapy target delineation, real-time monitoring and prognosis assessment [31]. Accurate understanding of tumour characteristics and stratification of HNSCC patients are prerequisites for precise treatment strategies selection. Radiomics has been recognized as a bridge between medical imaging and individualized medicine in cancer treatment [32]. Sophisticated spatial and temporal anatomical information and intrinsic phenotypes derived from imaging may favorably manipulate therapeutic efficacy and toxic reactions. These applications show promise in personalized radiotherapy protocols and are reported in the following sections.

### Radiomics to Predict Therapeutic Efficacy

#### Prediction of Treatment Outcomes

Mono-radiomics features (RFs), when integrated with clinical risk factors, have demonstrated the ability to predict a range of clinical outcomes in HNSCC. Depending on the imaging source, RFs derived from diagnostic CT or radiotherapy planning CT have shown predictive efficacy for OS as well as other endpoints, including progression free survival (PFS), local control (LC), regional control (RC), distant metastasis-free survival (DMFS), and disease-free survival (DFS). Multimodal imaging like ultrasound, MRI, and PET offer complementary RFs that can further enhance the accuracy of outcome prediction.

Several factors can be incorporated into model development and influence its predictive capability. RFs derived from multitemporal imaging (before, during, or after treatment) have demonstrated significant differences in predicting OS and tumour control. Moreover, RFs extracted from the same imaging timepoint but from different regions of interests (ROIs) may improve the prediction effectiveness. Lin et al. [33] compared the models developed by pre- and interim- radiotherapy imaging data from intratumoral and peritumoral ROIs, showing that RFs from intratumoral ROI during radiotherapy achieved the highest AUC, with OS at 0.79 and PFS at 0.82.

#### Prediction of Treatment Response

Multimodal imaging has been widely applied to monitor tumour response and assess treatment outcomes. Evidence indicates that radiomics-based models can be instrumental in predicting treatment response across various treatment modalities, including radical RT, CRT, and IC + CRT. The optimal predictive model for different endpoints appears to vary substantially and may be influenced by factors such as imaging modality, patient cohort and treatment strategy. Models developed by computing RFs extracted from GTV on planning CT indicated that incorporating additional data does not always enhance predictive accuracy. Bogowiez et al. [34] predicted the local control based on a radiomics model, with better CI of 0.73. The addition of clinical parameters marginally improved the model performance in the training cohort but not in the validation cohort. In addition to the primary tumour, treatment responses to metastatic lymph nodes can also be predicted using radiomics models.

Delta radiomics [35], which extracts quantitative imaging features from the same tumour or tissue at different treatment timepoints, is commonly employed to predict treatment response by computing the changes (delta, $\:{\Delta\:}$) of feature values. Sellami et al. [36] applied delta radiomics utilizing weekly CBCT imaging, calculating a series of absolute differences compared to baseline CBCT1, with CBCTn-CBCT1 (n ranging from 1 to 7, corresponding to each week of radiotherapy, and $\:{\Delta\:}$Feature = Feature_post - Feature_pre). They reported that one longitudinal feature, Neighbouring Gray Tone Difference Matrix (NGTDM) coarseness, changed significantly during the fourth week of radiotherapy and a combined model with clinical data obtained the highest accuracy, with a balanced accuracy of 0.67 in the testing cohort.

#### Prediction of Replanning

Conventional fractionated radiotherapy for HNSCC is a prolonged treatment process, typically spanning 6 to 7 weeks. The maximum partial response is estimated to reach 80% to 90% among HNSCC patients during radiotherapy [37], characterized by regression of either the primary tumour and/or positive cervical lymph nodes. Nevertheless, patients often suffer from oral mucositis, nausea, pharyngeal pain, and dysphagia, particularly when undergoing CRT with a platinum-based regimen, leading to significant weight loss over a short period of time. Therefore, anatomical changes can occur and impair precise radiation delivery. Re-planning facilitates the adaptation of primary target volumes and radiotherapy planning to accommodate current anatomical and positional changes [38]. Lam et al. [39] investigated the RFs from planning CT to predict ill-fitted thermoplastic masks (IfTMs) triggered replanning events in NPC patients undergoing CRT, and the radiomics mono-model yielded the highest AUC of 0.784 and 0.723 in two different centers. Iliadou et al. [40] compared the RFs from CTV and parotid glands regions from CBCT, showing the highest accuracy of 0.90 in predicting early volume deviations among patients who require planning adaptation. Nevertheless, the model efficiency deteriorates from 0.90 to 0.72 over the radiotherapy journey, again indicating that temporal tumor radiomics trajectories can quantify dynamic tumor changes.

### Radiomics to Predict Toxic Reaction

Head and neck cancer RT planning is characterized by a large number of organs at risk (OARs) that serve essential daily functions, including memory, eyesight, mastication, deglutition, and phonation. Comprehensive consensus guidelines have defined 25 OARs in the head and neck region, providing concise delineation of their anatomic boundaries [41]. High radiation doses to these functional structures can lead to severe radiation-induced adverse events (rAEs) and impair quality of life (QoL). Dosimetric data from RT planning dose-volume histograms (DVH) and three-dimensional dose distributions (dosiomics) are routinely employed to evaluate the risk of injury to OARs. Incorporating radiomics and radiation dose data can predict acute and late rAEs prior to RT delivery, thereby prompting radiation oncologists to re-assess RT plans and allow for individualized adjustments in dose distribution to normal tissues.

#### Salivary Glands

Xerostomia is the most prevalent rAEs among patients with HNC undergoing RT, with over 70% reporting symptoms such as sticky saliva and hyposalivation [42]. The QUANTEC group recommend sparing at least the ipsilateral parotid gland to a mean dose less than 20 Gy, or bilateral parotid glands less than 25 Gy, to prevent severe xerostomia [43]. Nevertheless, achieving this constraint can be challenging in practice, as the deep lobe of the parotid lies adjacent to level II LNs, which are commonly considered a high-risk zone for metastasis and are covered with a high tumoricidal dose. Predictive models often incorporate parotid and submandibular glands, along with their corresponding dose distributions derived from various MRI sequences, which have showed different predictive performance. Similarly, late xerostomia can persist for prolonged periods, and RFs from scans at different timepoints may provide additional insights into the longitudinal changes in salivary glands function.

#### Brain

Radiation-induced temporal lobe injury (RTLI) is characterized by cerebral necrosis after high-dose radiation exposure and represents a late radiotherapy toxicity, predominantly observed in NPC patients with advanced T-stage tumours that invade the skull base [44]. The Radiation Therapy Oncology Group (RTOG) 0225 [45] has constrained temporal lobes dose, recommending a maximum dose below 60 Gy and limiting 1% of the temporal lobe volume to doses not exceeding 65 Gy. Yang et al. [46] reported on a large group involving 5,599 NPC patients, integrating RFs from planning CT with dosimetric data to develop a dosiomics risk model for predicting RTLI, with a CI of 0.811, and stratified the patients according to a 3-year temporal lobe injury-free survival (98.2% vs. 89.4% in the validation cohort, *p* < 0.001). Radiomics-based models can also predict treatment responses to RTLI. Furthermore, the early prediction of responses to late rAEs is of valuable in a clinical context as it enables oncologists to intervene promptly to alleviate symptoms or potentially delay the onset of late adverse events. For those patients who exhibit minimal or no response to standard treatment, individualized treatment protocols can be drawn up.

#### Mucosa, Muscle and Bone

Mucositis typically manifests within the first two weeks of radiotherapy. It has been reported that over half of patients experience severe mucositis (grade ≥ 3) at the end of RT [47]. Agheli et al. [48] contoured the oral mucosa structures as ROIs and integrated dosimetric with clinical data to develop a model with a higher AUC of 0.917, indicating that incorporating functional structures and their corresponding radiation doses may enhance the performance of toxicity prediction models. Orofacial functions such as mastication, deglutition, vocalization, and facial expressions require the fine motor of function-related muscles. High radiation dose involved to certain muscle groups, such as pharyngeal and cricopharyngeal constrictor muscle (PCM/CPM), the masseter, lateral and medial pterygoids, and temporalis muscles, may induce a range of symptoms [49]. Some of these symptoms will be alleviated after completion of RT, while others, such as dysphagia [50] and trismus [51], may persist long-term. Sheikh et al. [52] and Paetkau et al. [53] extracted RFs from post-treatment CT scans to predict acute (within 3 months) and late (12 months) dysphagia, respectively, and demonstrated that dosiomics features hold promise in predicting swallowing function. Thor et al. [54] utilized MRI-derived RFs to predict late trismus - they contoured several masticatory muscles and suggested different optimal models for each muscle. They indicated that the mean irradiation dose to masseter and medial pterygoids can achieve better AUCs of 0.85 and 0.77, respectively.

Osteoradionecrosis (ORN) is relatively uncommon in the era of intensity-modulated radiation therapy (IMRT), patients are routinely referred to oral surgery department for assessment and preventive treatment before RT. However, mandibular injury can occur if it is covered in a high-dose target region, leading to necrosis, tooth loss, infection, and failure to heal [55]. Barua et al. [56] compared RFs from ORN and non-ORN submandible volumes, and developed a model by the Multivariate Functional Principal Component Analysis (MFPCA) approach to characterize the temporal trajectories at pre- and post-treatment (2- and 6-month) timepoints of RFs, achieving an AUC of 0.74. Remarkably, in specific anatomical locations, such as the skull base and vertebrae, biopsies are risky and difficult to implement. Hence, radiologists have to rely on imaging-based diagnosis to distinguish amongst post-radiotherapy changes, ORN, and bone metastasis.

#### Weight Management

Weight management is crucial for patients undergoing anti-cancer treatments. The nutritional status of patients affects treatment intensity, such as considerations for combined treatments and the dose of chemotherapy agents. For patients receiving RT [57], rapid loss of subcutaneous fat will alter the target area location, especially in bilateral neck LNs. A body weight loss exceeding 5% from the start of RT to week 8, or over 7.5% by week 12, is defined as critical weight loss (cWL) [58]. Langius et al. [59] reported that patients who experienced cWL had lower 5-year OS and disease-specific survival (DSS) compared to those without cWL during RT, with rates of 62% vs. 70% (*p* = 0.01) and 82% vs. 89% (*p* = 0.001), respectively. Cheng et al. [60] developed a clinical decision support system (CDSS) model for weight loss, which may assist physicians in predicting patients weight loss. Their study suggested that both the identification and probability of weight loss predicted by physicians could be improved with the aid of CDSS, with the AUC increased from 0.58 to 0.63 and from 0.56 to 0.69, respectively.

### Comparative Analysis and Key Findings

Models developed on multimodal and multitemporal omics are constantly emerging (Appendix, summarized in Tables 1, 2, 3 and 4). AI-predicted and AI-guided personalized radiotherapy should incorporate spatiotemporal dynamics. Radiomics features extracted from different ROI volumes and from different single timepoint images can play diverse roles in predicting clinical outcomes. Moreover, changes across different spatiotemporal dimensions (delta radiomics) warrant further investigation. Prediction performance varies across imaging modalities. However, adding more multimodal or multitemporal data does not necessarily improve predictive accuracy. Excessive covariates can be redundant and may interfere with selecting truly significant variables. Model performance is also contingent upon the algorithms employed. Fatima et al. [61] computed recurrence of metastatic LNs by three different approaches: Fisher’s linear discriminant (FLD), k nearest neighbors (kNN), and support vector machine (SVM). Their findings indicated that the fourth-week ultrasound delta-radiomics model developed by SVM achieved a higher AUC of 0.84. For toxicity prediction, optimal biomarkers are structure-specific. Slight variations in predictive efficacy have been observed across studies.

## Current Challenges and Limitation of Radiomics AI Models

AI models are only as good as their training data. The standardization of radiomics protocols is the key concern for widespread clinical implementation, as biases introduced by various processing details during radiomics analysis can influence generalizability and reproducibility of results [62]. Most studies in this field are retrospective, involving small cohorts and relying on internal validation within the same dataset. External validation from independent or multicentre datasets is crucial for a comprehensive evaluation of the models. Another limitation is the “black box” nature inherent in deep learning [63], which makes it challenging to comprehend the logic behind model predictions. Oncologists may distrust fully automated decision-making tools that lack explainability and transparency. Explainable AI (XAI) approaches, such as Gradient-weighted Class Activation Mapping (Grad-CAM), SHapley Additive exPlanation (SHAP), and Local Interpretable Model-agnostic Explanations (LIME), have been proposed to address these limitations to some extent. Furthermore, ethical challenges arise because radiomics data still contain sensitive information from patients, therefore, normative anonymization techniques must be proposed to protect patient identities.

## Outlook

Tumours are constantly changing whilst being treated, so static standard treatment has its limitations. Personalized radiotherapy, however, not only customizes treatment but also tends to monitor tumour response and adjust therapeutic schemes adaptively, rather than simply escalating or de-escalating from standard care. Standard treatment heavily relies on evidence-based medicine, such as meta-analysis, randomized controlled trials (RCTs), and well-designed retrospective studies. However, few prospective RCTs are registered to evaluate AI in HNSCC radiotherapy, with only single-arm or pilot studies. The RadiomicART (NCT05081531) trial [64] aims to combine ART with radiomics analysis to customize the radiotherapy based on the shrinkage of tumour volumes and surrounding OARs. The ARCHERY (NCT05653063) trial [65] will examine whether AI-based contouring and planning can meet sufficient quality standards for routine clinical use in cervical, head and neck, and prostate cancer. Another trial (NCT05979883) [66] also compares machine learning-assisted planning (MLAP) with standard treatment planning to determine potential clinical benefits regarding acute toxicity and patient reported-outcomes. At the present stage, each of these projects remains under investigation. The consensus on how AI applied in clinical practice is gradually improving. In 2020, the Standard Protocol Items: Recommendations for Interventional Trials-Artificial Intelligence (SPIRIT-AI) extension provided a checklist for clinical trial protocols evaluating interventions with an AI component [67]. In 2024, the European Society for Radiotherapy and Oncology (ESTRO) and American Association of Physicists in Medicine (AAPM) jointly drafted a cohesive guideline for the application and reporting of AI models in radiotherapy [68]. Future studies are expected to expand in various areas.

**Fig. 2:**
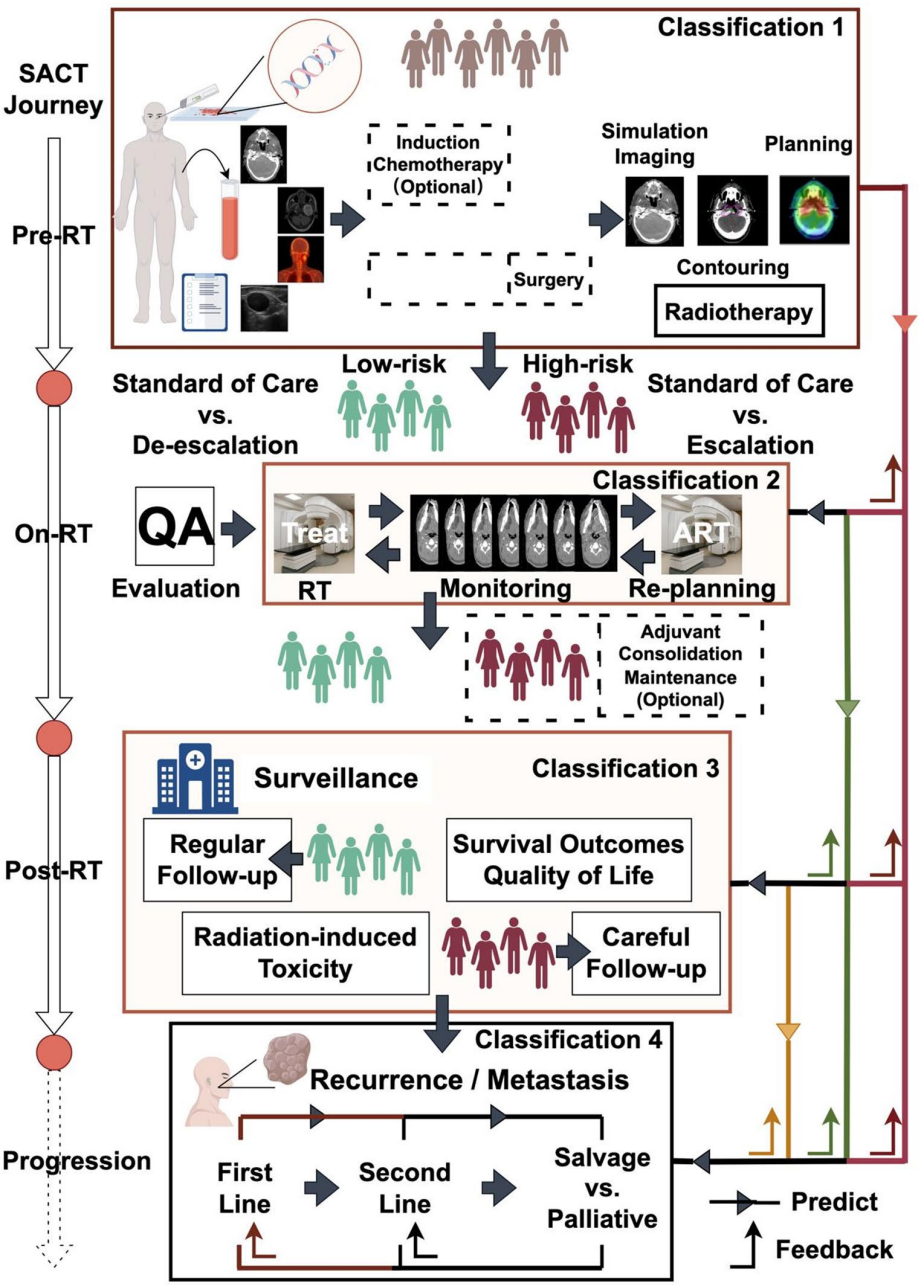
AI-driven framework for personalized radiotherapy. Classification 1: risk stratification guides treatment escalation or de-scalation. Classification 2: apply AI-driven monitoring and adaptive adjustments. Classification 3: tailor follow-up intensity and proactive interventions. Classification 4: predict risk and site of relapse to guide retreatment

Building upon previous research in personalized radiotherapy and current AI applications within this domain, we propose an AI-driven framework for personalized radiotherapy that outlines the potential roles of AI across the spectrum from pre-radiotherapy to salvage therapy. This framework introduces a four-step classification corresponding to different periods in HNSCC management, which may be considered for the design of future randomized controlled trials. (Fig. 2)

- The first classification pertains to the pre-radiotherapy phase, where comprehensive profiles, including clinical, radiological, histological, genetic, and dosimetric data, are integrated to predict treatment response, survival outcomes, recurrence patterns, radiation-induced toxicity, and replanning requests. Based on these predictive results, RT plans can be adjusted, and patients requiring plan modifications are identified for close monitoring throughout the RT process. The aim is to stratify patients into different risk categories. For those classified as high-risk, treatment escalation may involve expanding the target volume and increasing the RT dose prescription. Conversely, for low-risk patients, standard treatment or optional de-escalation strategies can be implemented.
- The second classification focuses on the on-treatment phase, utilizing imaging scans from linear accelerators such as CBCT and MVCT, or mid-treatment CT, MRI, and PET scans to feed the machine learning models. This classification emphasizes the analysis of radiomics changes (Delta radiomics) following the radiotherapy delivery. Survival outcomes and tumour control are re-evaluated, considering primary tumour shrinkage and the anatomical relationship between the target volume and normal tissues. Patients will be re-stratified, and AI-driven adaptive planning (adaptive radiotherapy) or real-time adjustments to the radiation beam settings (dynamic radiotherapy) are employed. Based on the aforementioned two stratifications, decisions are made regarding whether high-risk patients should receive consolidation therapy, maintenance therapy or surveillance.
- The third classification is applied during the post-treatment surveillance phase, where individualized follow-up schedules are offered based on the classification. For high-risk patients, intensive surveillance may be suggested compared to the standard surveillance interval. Similarly, follow-up imaging scans can be used to feed models for predicting late rAEs and corresponding therapeutic responses, enabling proactive interventions and the rapid development of customized treatment protocols to improve QoL.
- The fourth classification involves tumour recurrence and subsequent salvage therapy, computing multimodal imaging to predict the risk of tumour progression and the location of a second relapse, thereby enabling the adjustment of retreatment plans based on AI models rather than relying solely on palliative therapy. Re-irradiation will be re-evaluated and delivered in a cost-effective manner, based on the prediction of treatment response and dose-related toxicities from Classification 1 to 3, to maximize absolute benefit.

## Conclusion

AI holds substantial promise for personalizing radiotherapy across all stages. In the near future, integrating multiomics with big data may bridge the gap to individualized treatment. With advancements in sophisticated algorithms, predictions are expected to become more trustworthy and interpretable, driving treatment toward personalized precision oncology.

## Key References

- Lin CH, Yan JL, Yap WK, Kang CJ, Chang YC, Tsai TY, et al. Prognostic value of interim CT-based peritumoral and intratumoral radiomics in laryngeal and hypopharyngeal cancer patients undergoing definitive radiotherapy. Radiotherapy and Oncology. 2023;189. 10.1016/j.radonc.2023.109938.
  - This study demonstrated interesting results by comparing RFs from multispatial ROIs (intra- and peri-tumoral) during mid-radiotherapy rather than using conventional pre-treatment imaging.
- Beddok A, Orlhac F, Calugaru V, Champion L, Eddine CA, Nioche C, et al. 18 F -FDG PET and MRI radiomic signatures to predict the risk and the location of tumor recurrence after re-irradiation in head and neck cancer. European Journal of Nuclear Medicine and Molecular Imaging. 2023; 50:559–71. 10.1007/s00259-022-06000-7.
  - This study showed promising predictive results for recurrence after re-irradiation based on different imaging modalities of PET and MRI, although most of studies focus on prediction before initial treatment.
- Sellami S, Bourbonne V, Hatt M, Tixier F, Bouzid D, Lucia F, et al. Predicting response to radiotherapy of head and neck squamous cell carcinoma using radiomics from cone-beam CT images. Acta Oncol. 2022;61:73–80. 10.1080/0284186x.2021.1983207.
  - This study showed the prognostic value of multitemporal imaging, in which delta radiomics features were derived from weekly CBCT.
- Yang SS, OuYang PY, Guo JG, Cai JJ, Zhang J, Peng QH, et al. Dosiomics Risk Model for Predicting Radiation Induced Temporal Lobe Injury and Guiding Individual Intensity-Modulated Radiation Therapy. Int J Radiat Oncol Biol Phys. 2023;115:1291–300. 10.1016/j.ijrobp.2022.11.036.
  - This large cohort study of 5,599 patients demonstrated feasible results for predicting RTLI and how toxicity-prediction feedback can be applied to individualized radiotherapy.
- Berger T, Noble DJ, Yang ZL, Shelley LEA, McMullan T, Bates A, et al. Predicting radiotherapy-induced xerostomia in head and neck cancer patients using day-to-day kinetics of radiomics features. Physics & Imaging in Radiation Oncology. 2023;24:95–101. 10.1016/j.phro.2022.10.004.
  - This study showed interesting algorithms for predicting xerostomia by computing the day-to-day kinetics of RFs from MVCT.

## Supplementary Information

Below is the link to the electronic supplementary material.

Supplementary Material 1 (DOCX 88.0 KB)

## Data Availability

No datasets were generated or analysed during the current study.
