# Supplementary material for: Beyond Standard Radiotherapy: an AI-Driven Framework for Personalized Radiotherapy with a Four-Step Classification in Head and Neck Squamous Cell Carcinoma (HNSCC) Patients
Source: Curr Oncol Rep. 2025 Nov 1;27(11):1346–57. doi: 10.1007/s11912-025-01730-x (PMC12698768; doi:10.1007/s11912-025-01730-x)
Supplement: Supplementary file 1 — Supplementary Material 1 (DOCX 88.0 KB) [file 11912_2025_1730_MOESM1_ESM.docx]

Appendix: Summary of Studies in Model Comparison (***Section 5.3***)

As most studies in this field are retrospective, involving small cohorts and relying on internal validation within the same dataset, independent external validation has rarely been performed. Nevertheless, valuable similarities and differences can still be identified across these studies, even though retrospective evidence remains limited in strength. Therefore, these models are summarized in these supplementary tables.

Table 1. Radiomics to predict treatment outcomes ***(Section 5.1.1)***

Table 2. Radiomics to predict of treatment response ***(Section 5.1.2)***

Table 3. Radiomics to predict xerostomia ***(Section 5.2.1)***

Table 4. Radiomics to predict multiple radiation-induced adverse events ***(Section 5.2.2-3)***

| Study | Pts | Subsite | Survival Outcomes | Time | Imaging | Segmentation | Algorithms | Optimal Model  (Validation Cohort) |
| --- | --- | --- | --- | --- | --- | --- | --- | --- |
| Volpe et al. [1], 2023 | 105 | oropharynx | OS, LPFS | pre | CT | GTV | Cox, LASSO | CT + Clinical Model  OS CI 0.82, LPFS CI 0.86 |
| Zhai et al. [2], 2019 | 444 | H&N | LC, RC, DMFS,DFS | pre | planning CT | primary tumour  pathological LN | Cox, Logistic regression | CT + Clinical Model  LC CI 0.64, RC CI 0.80  DMFS CI 0.71, DFS CI 0.70 |
| Li et al. [3], 2022 | 145 | nasopharynx | OS | pre | planning CT | GTV + GTVn | LASSO-Cox | CT + Clinical Model  OS CI 0.849 |
| Ou et al. [4], 2017 | 120 | H&N | OS, PFS | pre | planning CT | primary tumour | Cox, PCA, Spearman | CT + p16 Model  OS AUC 0.78 |
| Franzese et al. [5], 2023 | 106 | H&N LN-positive | LRF, PFS, OS | pre | planning CT | GTVn | Cox,  Elastic Net | CT Model, LRF AUC 0.56  CT + Clinical Model  OS AUC 0.77, PFS AUC 0.71 |
| Tang et al. [6], 2021 | 188 | H&N | Death, Recurrence | pre | planning CT | GTV, PTV | DL-ANN, LOOCV | CT Deep Learning Model  Death GTV AUC 0.946  PTV AUC 0.925  Recurrence GTV AUC 0.96  PTV AUC 0.93 |
| Cozzi et al. [7], 2019 | 110 | H&N | OS, PFS, LC | pre | planning CT | GTV | Cox,  Elastic Net | CT Model OS CI 0.90,  PFS CI 0.80, LC CI 0.82 |
| Fatima et al. [8], 2021 | 51 | H&N LN-positive | Recurrence | interim | quantitative ultrasound | metastatic LN | FLD, kNN, SVM | US Delta Radiomics Model  Recurrence AUC week4 0.81 |
| Pei et al. [9], 2022 | 294 | nasopharynx | PFS | pre | MRI, T1WI+C, T2WI | tumour volumes | Cox, RSF | MRI + Clinical RSF Model  3y-PFS AUC 0.861  5y-PFS AUC 0.847 |
| Siow et al. [10], 2022 | 198 | hypopharynx | OS, PFS | pre | MRI, T1WI+C | tumour volumes of interest | LASSO-Cox | MRI + Clinical Model  OS iAUC 0.671,  PFS iAUC 0.675 |
| Beddok et al. [11], 2022 | 55 | H&N | LR/LEFS | pre-reRT | FDG-PET, MRI | reRT GTV | Spearman, PCA | MRI Model  Recurrence AUC 0.85  FDG-PET Model  LR location AUC 0.84 |
| Wang et al. [12], 2023 | 220 | H&N | OS | pre | 18F-FDG PET/CT  planning CT, DVH | GTV + GTVn | Cox, Spearman, LASSO-Cox | PET + CT + Dose Model  (3 different sets)  OS CI 0.873 / 0.759 / 0.835 |
| Sorensen et al. [13], 2020 | 29 | H&N | OS, DM, LC | pre and interim | FMISO-PET, FDG-PET | MTV | Cox | Radiomics features form  Pre/Mid-FMISO-PET |
| Carles et al. [14], 2021 | 35 | H&N | OS, PFS, LR, DM | pre and interim | FMISO-PET | HSV | WF, Spearman,  Cox | FMISO-PET Model  $\Delta$LGZE week5 LR AUC 0.80  $\Delta$CP week PFS, p = 0.009 |
| Zhang et al. [15], 2022 | 328 | H&N | LRRFS | post | FDG-PET, CT | GTV | mRMR, SVM, DA, Logistic regression, Cox | PET Model LRRFS AUC 0.90  CT + PET ± Clinical Model  LRRFS AUC 0.93/0.94 |
| Wu et al. [16], 2020 | 237 | H&N | LR | pre | FDG-PET, CT, planning dose | GTV  GTV + 1cm  GTV + 2cm | Cox, PCA, EasyEnsemble | Dosiomics + CT + PET Model  LR CI 0.66 |

Table 1. Radiomics to predict treatment outcomes (Section 5.1.1)

***Subsite:*** H&N, head and neck. LN, lymph node.

***Survival outcomes:*** OS, overall survival. LPFS, local progression free survival. LC, local control. RC, regional control. DMFS, distant metastasis-free survival. DFS, disease-free survival. PFS, progression free survival. DM, distant metastases. LRF, locoregional failure. LR, locoregional recurrence. LRRFS, locoregional recurrence free survival. LEFS, locoregional failure-free survival. reRT, re-irradiation.

***Segmentation:*** GTV, gross tumour volume. GTVn, gross tumour volume of lymph nodes. PTV, planning target volume. MTV, metabolic tumour volumes. HSV, hypoxic sub-volume.

***Algorithms:*** RSF, random survival forest. WF, wavelet band-pass filtering. DL-ANN, deep learning artificial neural networks. FLD, Fisher’s linear discriminant. kNN, k nearest neighbors. SVM, support vector machine. DA, discriminant analysis. PCA, principal component analysis.

***Optimal model:*** CI, concordance index. AUC, area under curve. $\Delta$, relative differences in radiomics feature. LGZE, low gray-level zone emphasis. CP, classification parameter. BA, balanced accuracy.

| Study | Pts | Subsite | Treatment Response | Time | Imaging | Segmentation | Algorithms | Optimal Model |
| --- | --- | --- | --- | --- | --- | --- | --- | --- |
| Bogowicz et al. [17], 2017 | 149 | H&N | CRT | pre | planning CT | GTVp | Cox, PCA  Logistic regression | CT Model  Local Control CI 0.73 |
| Kazmierska et al. [18], 2022 | 290 | H&N | (IC) + RT or CRT | pre | planning CT | GTVp | LASSO, Pearson correlation, MI,  Logistic regression | Clinical Model  Incomplete response AUC 0.78 |
| Xi et al. [19], 2022 | 272 | NPC | IC + CRT | pre and  post IC | MRI -T2WI, T1WI+C | primary tumour | mRMR, LASSO,  Logistic regression | MRI Delta Radiomics Model  TR AUC 0.910 |
| Yuan et al. [20], 2024 | 104 | NPC | (IC) + CRT | pre | MRI – T1WI(+C), T2WI, DWI, ADC | subregions within tumour area | K-means, LASSO, Pearson correlation, mRMR, Logistic regression, RF | ITH + Clinical Model  Early TR AUC 0.838 |
| Xu et al. [21], 2023 | 145 | NPC / LN | CRT | pre | MRI -T2WI, T1WI+C | the largest lymph nodes (+ 2mm) | LASSO, Spearman correlation, mRMR  Logistic regression | Peritumoral MRI Model  TR AUC 0.794 |
| Sellami et al. [22], 2021 | 93 | H&N | RT or CRT | interim | CBCT | GTVp | Spearman correlation,  Logistic regression | CBCT Delta + Clinical Model  TR BAcc 0.67 |

Table 2. Radiomics to predict of treatment response (Section 5.1.2)

***Subsite:*** NPC, nasopharyngeal cancer. LN, lymph node. H&N, Head and Neck.

***Treatment response:*** CRT, chemoradiotherapy. IC, induction chemotherapy. RT, radiotherapy.

***Segmentation:*** GTVp, gross tumour volumes of the primary tumour.

***Algorithms:*** MI, maximizing mutual information. mRMR, maximum relevance-minimum redundancy. RF, random forest.

***Optimal model:*** ITH, intratumor heterogeneity. TR, treatment response. BAcc, balanced accuracy.

| Study | Pts | Subsite | Xerostomia | Imaging | Segmentation | Algorithms | Optimal Model |
| --- | --- | --- | --- | --- | --- | --- | --- |
| Zhou et al. [23], 2022 | 52 | NPC | saliva amount reduction | planning CT, CT(simulator) | PGs | XGBoost, SHAP, Ridge regression, SVR, RF, Adaboost, decision tree | CT + DVH + Clinical Model  MSE 0.6994, R^2^ 0.9815 |
| Men et al. [24], 2019 | 784 | H&N | late xerostomia | planning CT | PGs, SMGs | 3D rCNN, Logistic regression | CT + Contour + Dosimetric  3D rCNN Model AUV 0.84 |
| Smith et al. [25], 2023 | 510 | H&N | late salivary hypofunction | planning CT | PGs | LKB, Spline-base, Neural network | Neural Network Model  AUC 0.75 - 0.83 |
| Sheikh et al. [26], 2019 | 266 | H&N | acute xerostomia (within 3 months) | CT, MRI(T1WI+C) | PGs, SMGs | LASSO,  Logistic regression | CT + MRI + DVH + Clinical Model AUC 0.68 |
| Dijk et al. [27], 2018 | 68 | H&N | late xerostomia  (12 months) | MRI(T1WI, TSE) | PGs | Pearson correlation, Logistic regression | MRI + Dose Model  AUC 0.83 |
| Calamandrei et al. [28], 2023 | 27 | OPC | xerostomia | MRI (T1WI+C, DWI, DCE-PWI) | PGs, SMGs | Shapiro-Wilk, parametric t-Student, Wilcoxon signed rank | PGs MRI Radiomics Features  Diagnostic Accuracy  AUC 0.727 |
| Berger et al. [29], 2023 | 117 | H&N | late xerostomia (6,12,24 months) | MVCT | PGs | Logistic regression | 6-m MVCT + DVH Model  AUC 0.69  12-m MVCT Model AUC 0.74  24-m MVCT Model AUC 0.86 |
| Abdollahi et al. [30], 2023 | 31 | H&N | late xerostomia | CT | PTV, PGs | LASSO | post CT, pre/mid CT Delta + Dosiomics, and DVH  Model AUC 0.89 |

Table 3. Radiomics to predict xerostomia (Section 5.2.1)

***Subsite:*** NPC, nasopharyngeal cancer. H&N, head and neck. OPC, oropharyngeal cancer.

***Imaging:*** TSE, turbo spin echo. DWI, diffusion-weighted imaging. DCE-PWI, dynamic contrast-enhanced, perfusion-weighted imaging. MVCT, mega-voltage CT image-guidance scans.

***Segmentation:*** PGs, parotid glands. SMGs, submandibular glands. PTV, planning target volume.

***Algorithms:*** XGBoost, eXtreme Gradient Boosting. SHAP, SHapley Additive exPlanations. SVR, support vector regression. 3D rCNN, three-dimensional residual convolutional neural network. LKB, Lyman-Kutcher-Burman model.

***Optimal model:*** MSE, mean square error. R^2^, coefficient of determination.

| Study | Pts | Subsite | Toxicity | Time | Imaging | Segmentation | Algorithms | Optimal Model |
| --- | --- | --- | --- | --- | --- | --- | --- | --- |
| Zhang et al. [31], 2020 | 242 | NPC | TL injury | Late | MRI (T1WI+C, T2WI) | Medial TL | relief mothed, RF | Last 1 MRI before RTLI  RF Model AUC 0.830 |
| Yang et al. [32], 2023 | 5599 | NPC | TL injury | Late | planning CT | Temporal lobes | Cox, LASSO, Logistic regression | Dosiomics + DVH + Clinical  Model CI 0.811 |
| Bao et al. [33], 2022 | 216 | NPC | TL injury | Late | MRI (T1WI+C, T2WI) | Temporal lobes | LASSO, Logistic regression | First Follow-up MRI + Clinical Model AUC 0.93 |
| Dong et al. [34], 2023 | 242 | NPC | Mucositis | Acute | CT, MRI (T1WI+C, T2WI), | GTVp  GTVn | RF, LR, GNB, XGBoost, SHAP | GTVp CT + MRI + Dosiomics GNB Model AUC 0.81±0.01 |
| Agheli et al. [35], 2024 | 49 | H&N | Mucositis | Acute | planning CT | oral mucosa structures | mRMR, RF | CT + Dosimetric + Clinical  RF Model AUC 0.917 |
| Sheikh et al. [36], 2019 | 136 | H&N | Dysphagia | Acute | CT | Swallow-related structures | Logistic regression | GLCM of salivary gland  Dose to swallow muscles |
| Paetkau et al. [37], 2024 | 87 | H&N | Dysphagia | Late  1-year | planning CT  surveillance CT | constrictor muscle | Random Forest, Pearson filter, K-Best | planning CT + Clinical Model  BA 0.71±0.23 |
| Thor et al. [38], 2017 | 10 | H&N | Trismus | Late  1-year | post MRI  (T1WI +C) | Masseter, Pterygoids(L/M) | Spearman correlation,  Logistic regression | Dmean to Masseter AUC 0.85  Dmean to MP AUC 0.77  GLCM of MP AUC 0.78 |

Table 4. Radiomics to predict multiple radiation-induced adverse events (Section 5.2.2-3)

***Subsite:*** NPC, nasopharyngeal cancer. H&N, head and neck cancer.

***Toxicity:*** TL, temporal lobe.

***Segmentation:*** GTVp, gross target volume of primary tumour. GTVn, gross target volume of lymph nodes. L, lateral. M, medial.

***Algorithms:*** RF, random forest. LR, logistic regression. GNB, Gaussian Naïve Bayes. XGBoost, extreme gradient boosting. SHAP, Shapley Additive Explanations. FPCA, functional principal component analysis.

***Optimal model:*** RTLI, radiation-induced temporal lobe injury. Dmean, the mean irradiation dose. MP, medial pterygoids. GLCM, Gray-Level Co-occurrence Matrix. BA balanced accuracy.

Reference

[1] Volpe S, Gaeta A, Colombo F, Zaffaroni M, Vincini MG, Pepa M, et al. CT-based radiomics for outcome prediction in oropharyngeal cancer patients treated with curative RT. Radiotherapy and Oncology. 2023;182:S1886–S7.

[2] Zhai TT, Langendijk JA, van Dijk LV, Halmos GB, Witjes MJH, Oosting SF, et al. The prognostic value of CT-based image-biomarkers for head and neck cancer patients treated with definitive (chemo-)radiation. Oral Oncol. 2019;95:178–86.

[3] Li XY, Chen H, Zhao FP, Zheng Y, Pang HW, Xiang L. Development of a Radiotherapy Localisation Computed Tomography-Based Radiomic Model for Predicting Survival in Patients With Nasopharyngeal Carcinoma Treated With Intensity-Modulated Radiotherapy Following Induction Chemotherapy. Cancer Control. 2022;29.

[4] Ou D, Blanchard P, Rosellini S, Levy A, Nguyen F, Leijenaar RTH, et al. Predictive and prognostic value of CT based radiomics signature in locally advanced head and neck cancers patients treated with concurrent chemoradiotherapy or bioradiotherapy and its added value to Human Papillomavirus status. Oral Oncol. 2017;71:150–5.

[5] Franzese C, Lillo S, Cozzi L, Teriaca MA, Badalamenti M, Di Cristina L, et al. Predictive value of clinical and radiomic features for radiation therapy response in patients with lymph node-positive head and neck cancer. Head Neck. 2023;45:1184–93.

[6] Fh T, Cyw C, Eyw C. Radiomics AI prediction for head and neck squamous cell carcinoma (HNSCC) prognosis and recurrence with target volume approach. BJR Open. 2021;3:20200073.

[7] Cozzi L, Franzese C, Fogliata A, Franceschini D, Navarria P, Tomatis S, et al. Predicting survival and local control after radiochemotherapy in locally advanced head and neck cancer by means of computed tomography based radiomics. Strahlenther Onkol. 2019;195:805–18.

[8] Fatima K, Dasgupta A, DiCenzo D, Kolios C, Quiaoit K, Saifuddin M, et al. Ultrasound delta-radiomics during radiotherapy to predict recurrence in patients with head and neck squamous cell carcinoma. Clinical and Translational Radiation Oncology. 2021;28:62–70.

[9] Pei W, Wang C, Liao H, Chen X, Wei Y, Huang X, et al. MRI-based random survival Forest model improves prediction of progression-free survival to induction chemotherapy plus concurrent Chemoradiotherapy in Locoregionally Advanced nasopharyngeal carcinoma. BMC Cancer. 2022;22:739.

[10] Siow TY, Yeh CH, Lin GG, Lin CY, Wang HM, Liao CT, et al. MRI Radiomics for Predicting Survival in Patients with Locally Advanced Hypopharyngeal Cancer Treated with Concurrent Chemoradiotherapy. Cancers. 2022;14.

[11] Beddok A, Orlhac F, Calugaru V, Champion L, Eddine CA, Nioche C, et al. 18F -FDG PET and MRI radiomic signatures to predict the risk and the location of tumor recurrence after re-irradiation in head and neck cancer. European Journal of Nuclear Medicine and Molecular Imaging. 2023;50:559–71.

[12] Wang B, Liu J, Zhang X, Wang Z, Cao Z, Lu L, et al. Prognostic value of (18)F-FDG PET/CT-based radiomics combining dosiomics and dose volume histogram for head and neck cancer. EJNMMI Res. 2023;13:14.

[13] Soerensen A, Carles M, Bunea H, Majerus L, Stoykow C, Nicolay NH, et al. Textural features of hypoxia PET predict survival in head and neck cancer during chemoradiotherapy. European Journal of Nuclear Medicine and Molecular Imaging. 2020;47:1056–64.

[14] Carles M, Fechter T, Grosu AL, Sörensen A, Thomann B, Stoian RG, et al. (18)F-FMISO-PET Hypoxia Monitoring for Head-and-Neck Cancer Patients: Radiomics Analyses Predict the Outcome of Chemo-Radiotherapy. Cancers (Basel). 2021;13.

[15] Zhang Q, Wang K, Zhou Z, Qin G, Wang L, Li P, et al. Predicting local persistence/recurrence after radiation therapy for head and neck cancer from PET/CT using a multi-objective, multi-classifier radiomics model. Front Oncol. 2022;12:955712.

[16] Wu AQ, Li YB, Qi MK, Lu XY, Jia QY, Guo FT, et al. Dosiomics improves prediction of locoregional recurrence for intensity modulated radiotherapy treated head and neck cancer cases. Oral Oncology. 2020;104.

[17] Bogowicz M, Riesterer O, Ikenberg K, Stieb S, Moch H, Studer G, et al. Computed Tomography Radiomics Predicts HPV Status and Local Tumor Control After Definitive Radiochemotherapy in Head and Neck Squamous Cell Carcinoma. Int J Radiat Oncol Biol Phys. 2017;99:921–8.

[18] Kaźmierska J, Kaźmierski MR, Bajon T, Winiecki T, Bandurska-Luque A, Ryczkowski A, et al. Prediction of Incomplete Response of Primary Tumour Based on Clinical and Radiomics Features in Inoperable Head and Neck Cancers after Definitive Treatment. J Pers Med. 2022;12.

[19] Xi Y, Ge X, Ji H, Wang L, Duan S, Chen H, et al. Prediction of Response to Induction Chemotherapy Plus Concurrent Chemoradiotherapy for Nasopharyngeal Carcinoma Based on MRI Radiomics and Delta Radiomics: A Two-Center Retrospective Study. Front Oncol. 2022;12:824509.

[20] Yuan J, Wu M, Qiu L, Xu W, Fei Y, Zhu Y, et al. Tumor habitat-based MRI features assessing early response in locally advanced nasopharyngeal carcinoma. Oral Oncol. 2024;158:106980.

[21] Xu H, Wang A, Zhang C, Ren J, Zhou P, Liu J. Intra- and peritumoral MRI radiomics assisted in predicting radiochemotherapy response in metastatic cervical lymph nodes of nasopharyngeal cancer. BMC Med Imaging. 2023;23:66.

[22] Sellami S, Bourbonne V, Hatt M, Tixier F, Bouzid D, Lucia F, et al. Predicting response to radiotherapy of head and neck squamous cell carcinoma using radiomics from cone-beam CT images. Acta Oncol. 2022;61:73–80.

[23] Zhou L, Zheng WJ, Huang SJ, Yang X. Integrated radiomics, dose-volume histogram criteria and clinical features for early prediction of saliva amount reduction after radiotherapy in nasopharyngeal cancer patients. Discover Oncology. 2022;13.

[24] Men K, Geng HZ, Zhong HY, Fan Y, Lin A, Xiao Y. A Deep Learning Model for Predicting Xerostomia Due to Radiation Therapy for Head and Neck Squamous Cell Carcinoma in the RTOG 0522 Clinical Trial. International Journal of Radiation Oncology Biology Physics. 2019;105:440–7.

[25] Smith DK, Clark H, Hovan A, Wu J. Neural network and spline-based regression for the prediction of salivary hypofunction in patients undergoing radiation therapy. Radiation Oncology. 2023;18.

[26] Sheikh K, Lee SH, Cheng Z, Lakshminarayanan P, Peng L, Han P, et al. Predicting acute radiation induced xerostomia in head and neck Cancer using MR and CT Radiomics of parotid and submandibular glands. Radiat Oncol. 2019;14:131.

[27] van Dijk LV, Thor M, Steenbakkers R, Apte A, Zhai TT, Borra R, et al. Parotid gland fat related Magnetic Resonance image biomarkers improve prediction of late radiation-induced xerostomia. Radiother Oncol. 2018;128:459–66.

[28] Calamandrei L, Mariotti L, Bicci E, Calistri L, Barcali E, Orlandi M, et al. Morphological, Functional and Texture Analysis Magnetic Resonance Imaging Features in the Assessment of Radiotherapy-Induced Xerostomia in Oropharyngeal Cancer. Applied Sciences-Basel. 2023;13.

[29] Berger T, Noble DJ, Yang ZL, Shelley LEA, McMullan T, Bates A, et al. Assessing the generalisability of radiomics features previously identified as predictive of radiation-induced sticky saliva and xerostomia. Physics & Imaging in Radiation Oncology. 2023;25.

[30] Abdollahi H, Dehesh T, Abdalvand N, Rahmim A. Radiomics and dosiomics-based prediction of radiotherapy-induced xerostomia in head and neck cancer patients. Int J Radiat Biol. 2023;99:1669–83.

[31] Zhang B, Lian Z, Zhong L, Zhang X, Dong Y, Chen Q, et al. Machine-learning based MRI radiomics models for early detection of radiation-induced brain injury in nasopharyngeal carcinoma. BMC Cancer. 2020;20:502.

[32] Yang SS, OuYang PY, Guo JG, Cai JJ, Zhang J, Peng QH, et al. Dosiomics Risk Model for Predicting Radiation Induced Temporal Lobe Injury and Guiding Individual Intensity-Modulated Radiation Therapy. Int J Radiat Oncol Biol Phys. 2023;115:1291–300.

[33] Bao D, Zhao Y, Li L, Lin M, Zhu Z, Yuan M, et al. A MRI-based radiomics model predicting radiation-induced temporal lobe injury in nasopharyngeal carcinoma. Eur Radiol. 2022;32:6910–21.

[34] Dong YJ, Zhang J, Lam S, Zhang XY, Liu AR, Teng XZ, et al. Multimodal Data Integration to Predict Severe Acute Oral Mucositis of Nasopharyngeal Carcinoma Patients Following Radiation Therapy. Cancers. 2023;15.

[35] Agheli R, Siavashpour Z, Reiazi R, Azghandi S, Cheraghi S, Paydar R. Predicting severe radiation-induced oral mucositis in head and neck cancer patients using integrated baseline CT radiomic, dosimetry, and clinical features: A machine learning approach. Heliyon. 2024;10:e24866.

[36] Sheikh K, Cheng Z, Lakshminarayanan P, Mathews L, Deek MP, McNutt TR, et al. Relationship of CT Radiomics and Dose Texture to Radiation-Induced Swallow Dysfunction in Head and Neck Cancer. International Journal of Radiation Oncology Biology Physics. 2019;105:S170–S1.

[37] Paetkau O, Weppler S, Quon HC, Tchistiakova E, Kirkby C. Developing and validating multi-omics prediction models for late patient-reported dysphagia in head and neck radiotherapy. Biomed Phys Eng Express. 2024;10.

[38] Thor M, Tyagi N, Hatzoglou V, Apte A, Saleh Z, Riaz N, et al. A Magnetic Resonance Imaging-based approach to quantify radiation-induced normal tissue injuries applied to trismus in head and neck cancer. Phys Imaging Radiat Oncol. 2017;1:34–40.
